# Green and Cost-Effective Synthesis of Tin Oxide Nanoparticles: A Review on the Synthesis Methodologies, Mechanism of Formation, and Their Potential Applications

**DOI:** 10.1186/s11671-021-03555-6

**Published:** 2021-05-28

**Authors:** Yemane Tadesse Gebreslassie, Henok Gidey Gebretnsae

**Affiliations:** 1 Department of Chemistry, College of Natural and Computational Science, Adigrat University, P.O. Box 50, Adigrat, Ethiopia; 2 African Chair in Nanoscience and Nanotechnology, College of Graduate Studies, UNESCO-UNISA, Muckleneuk ridge, PO Box 392, Pretoria, South Africa; 3 Nanosciences African Network, Materials Research Department, iThemba LABS, Cape Town, South Africa

**Keywords:** Tin oxide nanoparticles, Green synthesis, Antimicrobial activity, Photocatalytic activity

## Abstract

Nanotechnology has become the most promising area of research with its momentous application in all fields of science. In recent years, tin oxide has received tremendous attention due to its fascinating properties, which have been improved with the synthesis of this material in the nanometer range. Numerous physical and chemical methods are being used these days to produce tin oxide nanoparticles. However, these methods are expensive, require high energy, and also utilize various toxic chemicals during the synthesis. The increased concerns related to human health and environmental impact have led to the development of a cost-effective and environmentally benign process for its production. Recently, tin oxide nanoparticles have been successfully synthesized by green methods using different biological entities such as plant extract, bacteria, and natural biomolecules. However, industrial-scale production using green synthesis approaches remains a challenge due to the complexity of the biological substrates that poses a difficulty to the elucidations of the reactions and mechanism of formations that occur during the synthesis. Hence, the present review summarizes the different sources of biological entities and methodologies used for the green synthesis of tin oxide nanoparticles and the impact on their properties. This work also describes the advances in the understanding of the mechanism of formation reported in the literature and the different analytical techniques used for characterizing these nanoparticles.

## Introduction

In the last few decades, nanotechnology has emerged as a new area of research dealing with the synthesis, characterization, modification and utilization of nanomaterials for their tremendous application in areas like pharmaceutics, food industry, cosmetics, textile industry, medicine, optics, electronics, energy science and electrochemical applications [1–4]. Nanomaterials are materials having one dimension in a size range of 1–100 nm. The very large surface area-to-volume ratio and extremely small size of these materials may result in a completely new or enhanced electrical, optical, magnetic, catalytic, and antimicrobial activities as compared to their bulk materials [5–7]. Because of these unique properties, nanoparticles find application in various fields of modern science and engineering, such as nanomedicine, photocatalysis, biosensors, cleaning agents, and textile industry [1, 8]. Among the nanoparticles, tin(IV) oxide (SnO_2_) in particular has gained immense attention due to their versatile applications such as optoelectronic devices [9], solid-state gas sensors [10], electrodes for lithium-ion batteries [11], field emission displays [12], light-emitting diodes [13], catalysis [14], dye-based solar cells [15], medicine [16], photo-sensors and antistatic coatings [17].

In material science, SnO_2_ is considered as an oxygen-deficient n-type semiconductor, which crystallizes as the tetragonal rutile structure with lattice constants *a* = *b* = 4.7374 Å and *c* = 3.1864 Å. The unit cell consists of two sixfold coordinated tin and four three-fold coordinated oxygen atoms [18, 19]. A wide energy gap (3.6 to 3.8 eV), strong thermal stability (up to 500 °C), a high degree of transparency in the visible spectrum, strong chemical and physical interactions with the adsorbed species make SnO_2_ a promising candidate for a potential application in the lithium-ion batteries, sensors, catalysis, energy storage, glass coatings, medicine and environmental remediation [20–23]. SnO_2_ is used as a sensor to improve the response time and sensitivity owing to its high specific area, high chemical stability, low electrical resistance, and low density [24]. From the past few years, SnO_2_ was thoroughly explored for its applications in the solar cells [25] and gas sensors to detect the combustible gases such as CO, NO, NO_2_, H_2_S, and C_2_H_5_OH [26–29]. Due to the unique physicochemical properties and potential applications of nanoparticles (NPs), the scientific community has been developing several methods for producing nanoparticles. However, the chemical and physical methods used for the synthesis of metal and metal oxide nanoparticles are quite expensive and use toxic substances that are hazardous to the environment and human health [30]. In recent years, most researchers have changed their research interest toward the green synthesis of NPs because it has many advantages such as cost-effective, simple manufacturing procedure, reproducibility in production, and often results in more stable nanoparticles [31]. During the last decade, several studies on the green synthesis of SnO_2_ NPs were reported. However, no single review article is available in the literature that demonstrates the methodology of synthesis and mechanism of formation. Hence, this paper describes the green synthesis, mechanism of formation, characterization techniques, and potential applications of SnO_2_ NPs.

## Green Synthesis of Tin Oxide Nanoparticles

SnO_2_ NPs are synthesized by various physical, chemical, and green methods. The chemical methods include sol–gel, hydrothermal, precipitation, mechanochemical method, microemulsion, and so forth [31–37]. Among the chemical methods, the most widely used technique is the sol–gel synthesis, which utilizes tin precursor salt and chemical reagents that regulate the formation of the tin-containing gel. After that, the gel is exposed to heat treatment under temperatures up to 800 °C to obtain SnO_2_ NPs [32, 38]. Chemical stabilizers and capping agents, such as oxalic acid or ethylene glycols, can be added during the synthesis of SnO_2_ NPs to control the size and forbid agglomeration of the nanoparticles [32, 39]. A solution pH, the concentration of chemicals, reaction time, and calcination temperature can also influence the size and morphology of nanoparticles [31, 34–37]. The aforesaid methods of synthesizing SnO_2_ NPs utilize various perilous chemical reagents, solvents, and surfactants, which create a serious threat to the environment and human health [4, 30].

SnO_2_ NPs can also be synthesized by physical techniques such as spray pyrolysis, thermal oxidation, chemical vapor deposition, laser ablation, and ultrasonication [40–44]. Among these methods, laser ablation is considered a cost-effective and simple method for synthesizing metal and metal oxide nanoparticles in liquid [44, 45]. In contrast to the other conventional methods, this method does not require capping/reducing agents, high temperature, or high pressure, and allows us to produce nanoparticles of high purity [44, 45]. The variation in the parameters of the pulses applied from the laser beam and the ablation time are important parameters that define the particle size, morphology, and surface chemistry of the nanoparticles [44]. However, most of the physical methods require complex equipment, high energy, and skilled man power [46]. Therefore, it is highly important to develop synthesis methods that are eco-friendly, cheap, efficient, and that works at ambient conditions. One such solution is a green synthesis, and many researchers have developed a green chemistry approach for synthesizing tin oxide nanoparticles. In the green synthetic strategy, biological entities like plant extract, microorganisms or other green sources could be used as an alternative to the conventional physical and chemical methods [47]. These days, the biologically inspired methods of synthesis are also known as green synthesis because they go in agreement with the twelve principles of green chemistry [48]. Some of the distinct advantages that biological synthesis has over physical and chemical methods are (a) clean and environmentally friendly method, as nontoxic chemicals are used, (b) the use of renewable sources, (c) the active biological components like enzyme itself as well as phytochemicals acts as reducing and capping agent, thereby minimizing the overall cost of the synthesis process, (d) external experimental conditions like high pressure and temperature are not required, causing significant energy savings [49, 50].

In the last decade, the interest in synthesizing SnO_2_ NPs via the biological method has increased considerably, because the process is more reliable, eco-friendly, cost-effective, low input-high yield, and simple procedures without causing any adverse effect on the environment. A variety of biological substrates such as plant extracts, bacteria, and natural biomolecules have been successfully employed for green synthesis of SnO_2_ NPs. The phytochemicals of various plants and enzymes from bacteria are primarily responsible for the green synthesis. The active compounds present in the green sources also play as reducing, capping, and stabilizing agent during the synthesis. The desired NPs are often obtained after calcination or annealing at specified temperatures [51–54].

### Plant-Mediated Synthesis of Tin Oxide Nanoparticles

**Fig. 1:**
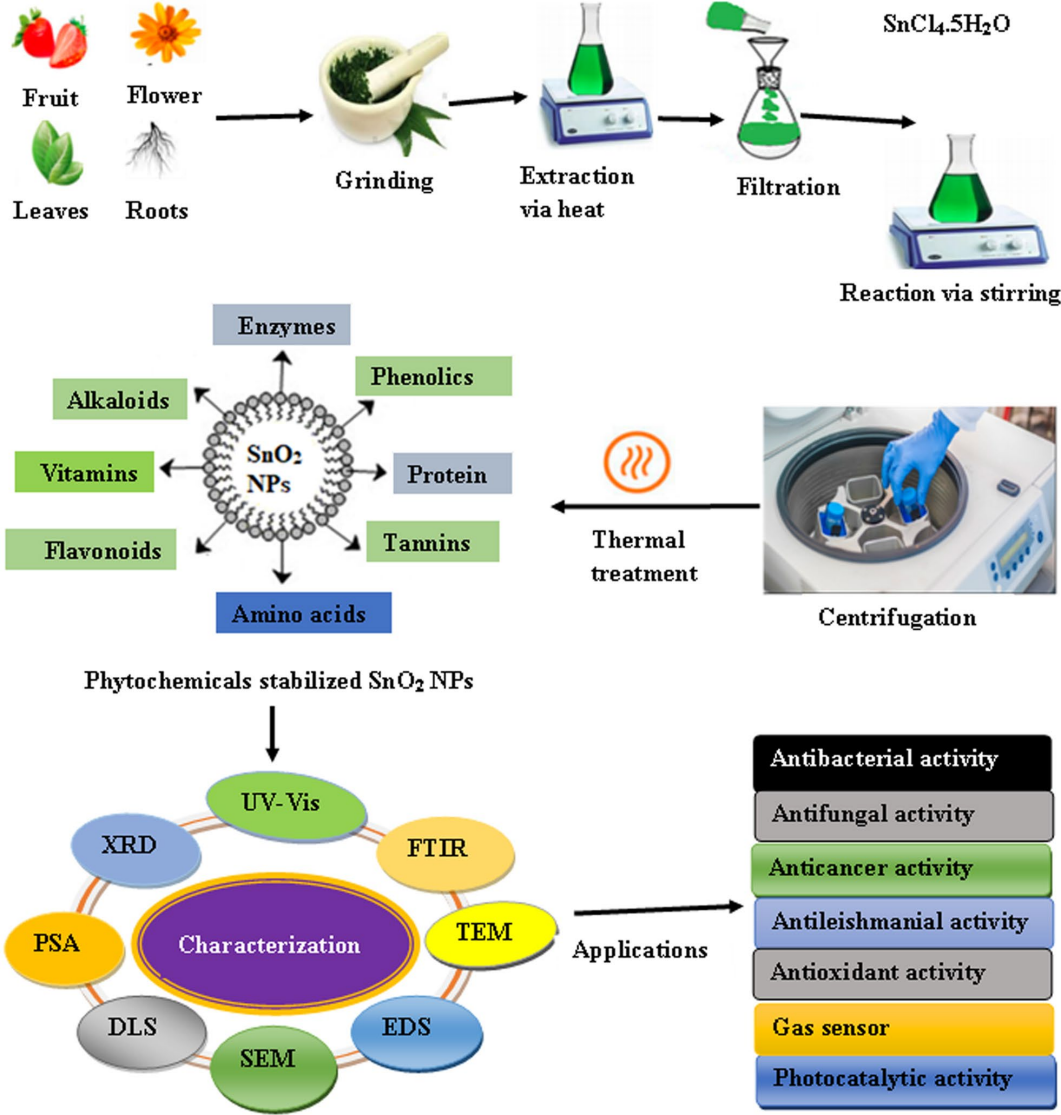
A schematic diagram of plant extract-mediated synthesis of SnO_2_ NPs

Plant-mediated synthesis has become the best platform of synthesis over conventional physicochemical methods, because it is free from toxic chemicals and provides natural capping as well as reducing agents. Moreover, it is easy and eco-friendly and gives quantity-enriched product free of impurities. In this method, there is no need to use high temperature, high pressure, and costly equipment. Additionally, plant-mediated synthesis leads to large-scale production of more stable nanoparticles with varying shapes and sizes [55, 56]. Extracts of a large number of parts of different plant species have been utilized for the green synthesis of SnO_2_ NPs. In general, the plant-mediated synthesis of SnO_2_ NPs is a very simple process in which a thin salt is added to the extract previously prepared. After the reaction, the solution is subjected to centrifugation. Finally, the pellets are then submitted to thermal treatment followed by characterization using various analytical techniques such as Fourier transform infrared spectroscopy (FTIR), X-ray diffractometer (XRD), energy-dispersive X-ray analysis (EDS), scanning electron microscopy (SEM), transmission electron microscopy (TEM), particle size analyzer (PSA), and dynamic light scattering (DLS). UV–visible spectroscopy (UV–Vis) is used to monitor the formation of nanoparticles. The detailed protocol for the plant-mediated synthesis of SnO_2_ NPs is schematically illustrated in Fig. 1. Diallo et al. reported the green synthesis of SnO_2_ NPs by using *Aspalathus linearis* and tin chloride pentahydrate (SnCl_4_·5H_2_O) as a precursor [51]. The salt was dissolved in the plant extract, and the formation of a white deposit was observed after 10 min. The white deposit was collected after the centrifugation process and was dried at about 80 °C. The powder was annealed at various temperatures for about 4 h and subjected to various analytical techniques like high-resolution transmission electron microscopy (HR-TEM), EDS, XRD, and X-ray photoemission spectroscopy (XPS). The NPs were quasi-spherical in shape with an average size in the range of 2.5–11.40 nm. It was observed that the particle size and crystalline nature of the NPs increase with increasing the annealing temperature. Moreover, the bioactive components like aspalathin, nothofagin, and aspalalinin present in the plant extract act as both chelating and reducing agents. *Camellia sinensis* leaf extract was also used to synthesize SnO_2_ NPs [53]. The polyphenols present in the leaf extract act both as stabilizing and as capping agents. High-resolution scanning electron microscopy (HR-SEM) and XRD analysis revealed spherical shape SnO_2_ NPs with size in the range of 5–30 nm. Furthermore, the bandgap of the NPs was found to decrease with increasing annealing temperature. The *Catunaregam spinosa*-mediated green synthesized SnO_2_ NPs showed excellent photocatalytic activity against Congo red dye [57]. The biosynthesized NPs are spherical in shape and an average size of 47 nm.

The leaf extract of *Aloe barbadensis miller* has been used to synthesize SnO_2_ NPs from SnCl_2_·2H_2_O as a precursor [58]. The resultant NPs was found to be spherical with an average size varying from 50 to 100 nm. Moreover, the synthesized nanoparticles exhibited excellent antibacterial activity against *S. aureus* and *E. coli.*

In another study, the green synthesis of SnO_2_ NPs was done by an efficient and cheap method using *Plectranthus amboinicus* leaf extract and SnCl_2_·2H_2_O as starting material [59]. The plant extract acts as a reducing and stabilizing agent. The resulting NPs were characterized by SEM, EDX, XRD, and PSA. The NPs were tetragonal in shape with an average particle size of 63 nm. Furthermore, the authors revealed that the biosynthesized NPs exhibited greater photocatalytic activity against Rhodamine B as compared to the commercial SnO_2_. Green synthesis of SnO_2_ NPs was reported using *Nyctanthes arbor-tristis* (Parijataka) flower extract [60]. As it is evident from SEM and PSA analysis, the synthesized nanoparticles exhibited the morphology of fine granules with tiny agglomeration stage and average grain size of 2–8 nm. The study also explored the hydrolysis and capping potential of the plant extract.

The green synthesis of SnO_2_ NPs was carried out by a low-cost and eco-friendly process using *Psidium guajava* leaf extract [61]. The UV–Vis result revealed a surface plasmon resonance peak at 314 nm, confirming the formation of SnO_2_ NPs. Moreover, the NPs were spherical with size ranging from 8 to 10 nm. The study also showed the NPs exhibit 90% degradation of reactive yellow 186 within 180 min under sunlight irradiation. Bhosale et al. [62] identified the leaf extract of *Calotropis gigantea* as a natural source for the synthesis of SnO_2_ NPs. The secondary metabolites of the extract act as both stabilizing and capping agent in the conversion of tin chloride to SnO_2_ NPs. The authors suggested that the NPs were spherical and an average size between 30 and 40 nm. The synthesized NPs degrade the methyl orange dye up to 80%, within 120 min. Singh et al. [63] reported the biosynthesis of SnO_2_ NPs using *Piper betle* leaf extract. SEM and TEM analysis revealed the formation of spherical NPs with an average size of 8.4 nm. The NPs degrade the reactive yellow 186 in a pseudo-first order with an efficiency of 92.17%. Additionally, the NPs showed excellent selectivity toward the removal of reactive yellow 186 as compared to reactive red 120 and reactive green 119. In the literature, different plants and plant extracts have been used for the preparation of SnO_2_ (Table 1). From the table, it can be seen that the nature of plant species and the reaction conditions affect the size and shape of SnO_2_ NPs.

**Table 1:** Plant extracts used for the synthesis of SnO_2_NPs with their shape, size and brief experimental condition

Plant (part)	Precursors	Synthesis conditions	Shape	Size	Applications	Ref
*Annona squamosa* (peel)	SnCl_2_·2H_2_O	Reaction: 60 °C, 3 h Drying: 160 °C, 1 h	Spherical	27.5 nm	Cytotoxic activity	[64]
*Aspalathus linearis* (leaf)	SnCl_4_·5H_2_O	Drying: 80 °C, Annealing: 400–900 °C, 4 h	Quasi-spherical	2.5–11.40 nm	Efficient photocatalyst against methylene blue, congo red, eosin Y	[51]
*Brassica oleracea L. var. botrytis* (leaf)	SnCl_2_·2H_2_O	Reaction: 60 °C, 6 h Drying: 75 °C, Annealing: 300–450 °C, 4 h	Spherical	3.1–6.34 nm	Excellent photocatalyst against methylene blue	[52]
*Camellia sinensis* (leaf)	SnCl_2_	Reaction: 80 °C, 30 min Drying: 80 °C, Annealing: 200–800 °C	Spherical	5–30 nm	–	[53]
*Calotropis gigantean* (leaf)	SnCl_2_·5H_2_O	Drying: microwave oven; Calcination: 400 °C, 3 h	Spherical	30–40 nm	Photocatalytic activity against methyl orange dye	[62]
*Catunaregam spinosa* (root)	SnCl_2_	Reaction: 60 °C, 2 h Annealing: 450 °C, 2 h	Spherical	47 nm	Efficient photocatalyst against congo red dye	[57]
*Cyphomandra betacea* (fruit)	SnCl_2_	Reaction: 80 °C, 3 h Annealing: 300 °C, 3 h	Spherical	20–50 nm	Excellent photocatalytic activity against methylene blue	[65]
*Daphne alpina* (leaf)	SnCl_4_·5H_2_O	Reaction: 60 °C, Drying: 120 °C	Elongated shape	19–27 nm	As adsorbent for Cd^2+^ ions	[66]
*Lisea cubeba* (fruit)	SnCl_2_·2H_2_O	Reaction: room temp., 10 min, Drying: 50 °C, 2 h;	Irregular	30 nm	Antioxidant activity Excellent photocatalytic activity against congo red	[67]
*Persia Americana* (seed)	SnCl_2_	Reaction: 60 °C, 12 h; Calcination: 300 °C, 2 h	Flake-like	4 nm	Excellent photocatalytic activity against phenolsulfonphthalein dye	[68]
*Piper betle* (leaf)	SnCl_2_·2H_2_O	Heating: 60 °C, 4 h Calcination: 400 °C, 4 h	Spherical	8.4 nm	Excellent photocatalytic activity against reactive yellow 186 dye	[63]
*Piper nigrum* (seed)	SnCl_2_·2H_2_O	Drying: 60 °C, Calcination: 300–900 °C, 1 h	Tetragonal	5–30 nm	Cytotoxic activity	[69]
*Pruni* *spinosae* flos	SnCl_2_·2H_2_O	Reaction: 80 °C,1 h	Spherical	9 nm	Biocidal against bacteria and fungi	[70]
*Psidium guajava* (leaf)	SnCl_4_	Stirring: 60 °C, 4 h Calcination: 400 °C, 4 h	Spherical	8–10 nm	Photocatalytic activity against reactive yellow 186	[61]
*Saccharum officinarum* (stem)	SnCl_2_·2H_2_O, AgNO_3_,	Reaction: 100 °C, 4 h Drying: 60 °C, Calcination: 200 °C, 400 °C, 2 h	Spherical	8–10 nm	Antibacterial activity against *P. aeruginosa*, *E. coli*, *B. subtillis* and *S. pneumonia* Antioxidant activity	[71]
*Trigonelle foenum-graecum (*seed*)*	SnCl_4_·5H_2_O	Reaction: room temp., 5 min; Annealing: 300–900 °C, 1 h	Spherical	2.2–3.3 nm	Antibacterial activity against E. coli Antioxidant activity against DPPH	[72]

### Bacteria-Mediated Synthesis of Tin Oxide Nanoparticles

Microbes are important nano-factories that gain immense potential as eco-friendly and cost-effective tools, avoiding toxic chemicals and the high energy needed by physicochemical synthesis. Various microbes such as bacteria, fungi, and yeast have been used for the synthesis of metal and metal oxide nanoparticles either intracellularly or extracellularly. Intracellular synthesis involves transporting the metal ions into the microbial cell and the formation of the NPs in the presence of the enzyme, coenzymes, and other biomolecules inside the cell. In extracellular synthesis, the metal ions are trapped on the surface of the microbial cell. The enzymes and proteins available at the surface reduce the metal ions and are responsible for providing stabilization to the NPs [73]. However, the extracellular synthesis is more advantageous compared to the intracellular pathway because it can be used to fabricate large quantities of NPs and eliminates various steps of synthesis required for the recovery of NPs [74].

Biological synthesis utilizing bacteria is green and cost-effective approach compared to chemical synthesis. However, this method has several drawbacks: (a) screening of the microbes is a time-consuming process, (b) requires careful monitoring of the culture broth and the entire process, and (c) is difficult to control the size and morphology of the NPs. Not all bacteria can synthesize NPs due to their intrinsic metabolic process and enzyme activities. Therefore, careful selection of an appropriate bacteria is necessary to produce NPs with well-defined size and morphology [74]. For instance, a study conducted by Srivastava and Mukhopadhyay [54] reported a low-cost, green, and simplest procedure for the synthesis of SnO_2_ NPs using fresh and clean *Erwinia herbicola* bacterial cells and an aqueous tin(II) chloride solution. The synthesized SnO_2_ NPs were mostly spherical with size in the range of 10 to 42 nm. It was reported that the bacterial protein and other biomolecules serve as a reducing and stabilizing agent during the synthesis of SnO_2_ NPs. These biomolecules also helped in controlling the size and aggregation of SnO_2_ NPs.

### Biomolecules and Other Green Source-Mediated Syntheses of Tin Oxide Nanoparticles

Apart from the plant- and bacterial-mediated synthesis of SnO_2_ NPs, researchers have developed a nontoxic, environmentally benign, and green chemistry approach by utilizing other biomolecules such as amino acids, vitamins, enzymes, and sugars (Table 2). Yang et al. [75] synthesized SnO_2_ NPs using a low-cost and environmentally friendly method using vitamin C (ascorbic acid), a naturally available biomolecule. TEM analysis showed the formation of spherical NPs with an average size around 30 nm. Vitamin C acts as both a capping and a reducing agent during the synthesis. The study suggested the vitamin C capped on the surface of SnO_2_ NPs decreased the oxidative stress caused by SnO_2_ NPs on the cells, leading to a reduced weight loss in neonatal mice. Spherical SnO_2_ NPs within the average particle size of 13 nm was prepared using carbohydrate (starch) [76]. It was suggested that the carbohydrate acts as a template, which could bind several metal cations through their functional groups, and hence, a uniform dispersion of the cations was observed. In another study, remnant water collected from soaked Bengal gram beans (*Cicer arietnum* L.) was employed for synthesizing un-doped SnO_2_, and Ni, Fe, and Au-doped SnO_2_ NPs [77–79]. The authors suggested that the pectin in the extract was responsible for the synthesis of the SnO_2_ NPs. The average crystallite size of the spherically shaped un-doped SnO_2_, Ni-doped SnO_2_, and Au-doped SnO_2_ NPs was found to be 11 nm, 6 nm, and 25 nm, respectively.

**Table 2:** Biomolecules or other green sources used for the synthesis of SnO_2_ NPs with their shape, size, and brief experimental condition

Biomolecules	Precursors	Synthesis conditions	Shape	Size	Applications	Ref
Ascorbic acid (Asc)	SnCl_2_·2H_2_O	Reaction: 80 °C , 8 h Calcination: 300 °C , 2 h	Spherical	30 nm	Bodyweight effect on neonatal mice	[75]
Arginine	SnCl_2_·2H_2_O	Irradiation: thirty 10 s 300 W shots on microwave oven Drying: 70 °C	Spherical	4–5 nm	Efficient photocatalyst in the degradation of methylene blue dye	[81]
Aspartic acid and glutamic acid	SnCl_2_·2H_2_O	Irradiation: thirty 10 s 300 W shots on microwave oven Drying: 70 °C	Spherical	1.6–2.6 nm	Excellent photocatalyst against Rose Bengal and Eosin Y	[82]
Glycine	SnCl_2_·2H_2_O, glycine	Reaction: 100 °C , 4 h Drying: 60 °C, Calcination: 200–600 °C , 2 h	Spherical	6–33 nm	–	[83]
L-lysine	SnCl_2_·2H_2_O	Reaction: 100 °C , 3 h Drying: 60 °C Calcination: 200 -500 °C , 2 h	Spherical	4–17 nm	Exceptional photocatalyst against triphenylmethane dyes An excellent catalyst for the reduction of 4-nitrophenol to 4-aminophenol	[84]
Tyrosine	SnCl_2_·2H_2_O	Reaction: 100 °C , 4 h Drying: 60 °C Calcination: 500 °C , 2 h	Tetragonal	15–20 nm	Photocatalytic activity against Violet 4 BSN dye	[85]
Sugarcane juice	SnCl_2_·2H_2_O	Irradiation: thirty 10-s shots on microwave oven Calcination: 200 °C	Spherical	2.5–4.5 nm (TEM)	An excellent catalyst for the reduction of p-nitrophenol Efficient photocatalyst for degradation of methylene blue and Rose Bengal	[86]

Another study has shown a green approach to synthesize SnO_2_ NPs using an eggshell membrane (ESM) [80], a natural bio-waste from the chicken eggshell. It was suggested that the ESM constituents biomolecules such as uronic acid and saccharides containing the aldehyde moieties, which act as a reducing agent during the synthesis. Morphological analysis showed the formation of rod, hexagonal, and spherical shape of SnO_2_ NPs with a particle size in the range 13–40 nm.

Different amino acids like glycine, arginine, aspartic acid, lysine, and tyrosine [81–85] have been used for the green synthesis of SnO_2_ NPs due to their good capping or complexing agents. Amino acid-mediated synthesis eliminates the use of toxic chemicals during the synthesis. Spherical SnO_2_ NPs were synthesized using arginine [81]. The morphological studies suggest that the synthesized SnO_2_ NPs were spherical with an average size in the range of 4–5 nm. Bhattacharjee et al. [83] used glycine to produce SnO_2_ NPs from stannous chloride. It was suggested that the NPs formed at 200 °C, 400 °C, and 600 °C are spherical, polycrystalline, and monodispersed in nature with an average size of 6, 16, and 33 nm, respectively. Further, the nanoparticle obtained at 400 °C was luminescent. Similarly, Begum et al. [84] demonstrated the synthesis of tetragonal rutile structure SnO_2_ NPs using L-lysine with size in the range of 4–17 nm.

## Mechanism of Formation of Tin Oxide Nanoparticles Through Green Synthesis

**Fig. 2:**
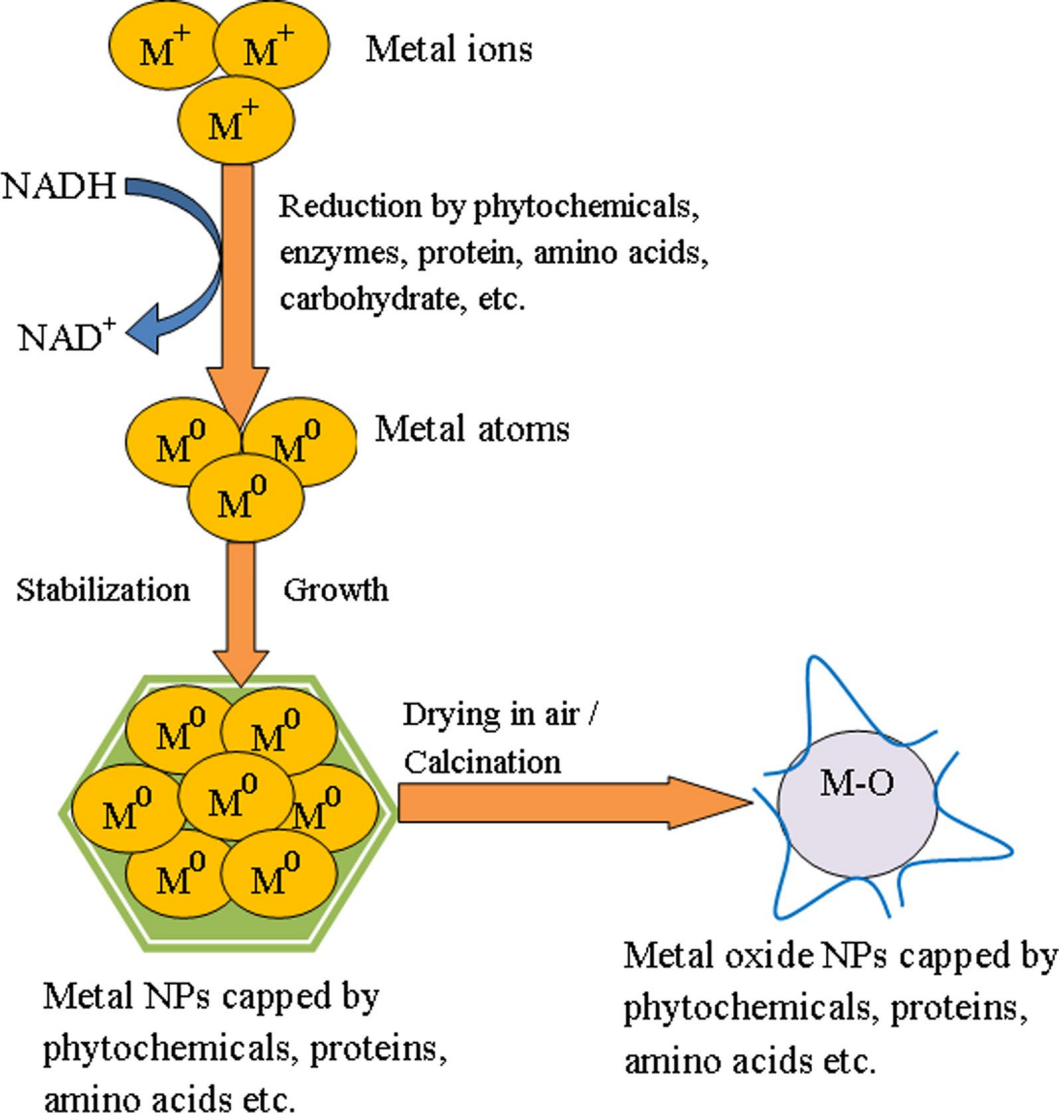
Diagram showing the possible mechanism for the biologically mediated synthesis of metal oxide (SnO_2_) NPs

In the last few years, different plant extracts, microorganisms, and other biological derivatives have been used in the green synthesis of metal and metal oxide NPs. Studies suggested that the secondary metabolites such as phenols, flavonoids, tannins, saponins, terpenoids, and carbohydrates present in the plant extract play a significant role by acting as both reducing and stabilizing agents in the synthesis of metal or metal oxide NPs [87]. Additionally, microbes-mediated synthesis can be attained either intracellularly or extracellularly according to the location where the NPs are formed. In extracellular synthesis, bioreduction occurs on the surface of the microbial cells in the presence of the enzymes available at the surface. Furthermore, in intracellular biosynthesis, metal ions are transported into the microbial cell, and the NPs easily form there in the presence of enzymes inside the cell [73]. Selvakumari et al. [53] utilized *Camellia sinensis* extract for the biosynthesis of SnO_2_ NPs. They demonstrated from their studies that the polyphenolic compounds (epicatechin, epigallocatechin, epicatechin gallate, and epigallocatechin gallate) present in the extract acts as both capping and stabilizing agents. The SnO_2_ NPs formed by this method consists of some major steps, including (i) reduction of Sn^2+^ to Sn^0^, (ii) the reducing effect of phenolic compounds (–OH) of extract to form Sn species, and (iii) thermal transformation of the Sn species into the SnO_2_ NPs. In the SnO_2_ NPs synthesized using *Calotropis gigantean* leaves extract, polyphenolic compounds are responsible for the biochemical transformations of tin ions [62]. Spherical SnO_2_ NPs of size in the range 3.62–6.34 nm were synthesized using Cauliflower extract [52]. It was suggested that the polyphenols and the flavonoids of the extract coordinate with the metal ions, resulting in the formation of Sn(OH)_2_ intermediates, which upon calcination leads to the formation of SnO_2_ NPs. In another study, Srivastava and Mukhopadhyay [54] reported both intracellular and extracellular synthesis of spherical SnO_2_ NPs by the enzymes secreted by bacterium *E. herbicola*. In this method, the Sn^2+^ ions are trapped by the extracellular enzymes secreted by the bacterial cell or by membrane-associated proteins on the cell surface, and the reduction was initiated by dehydrogenase enzymes. The Sn^2+^ ions are reduced by gaining two electrons, and a molecule of NAD^+^ is oxidized to form NADH, which finally results in the production of extracellular Sn NPs. The biosynthesized Sn nanoparticles are then oxidized by the oxygen present in the solution, which leads to the formation of SnO_2_ NPs. FTIR analysis revealed the presence of protein-like molecules on the surface, which provide natural support and stability of the SnO_2_ NPs. Furthermore, amino acids play as capping or complexing agents, thereby minimizing the use of toxic chemicals during the synthesis. The possible mechanism for the green synthesis of SnO_2_ NPs is shown in Fig. 2:

## Characterization of Tin Oxide Nanoparticles

**Fig. 3:**
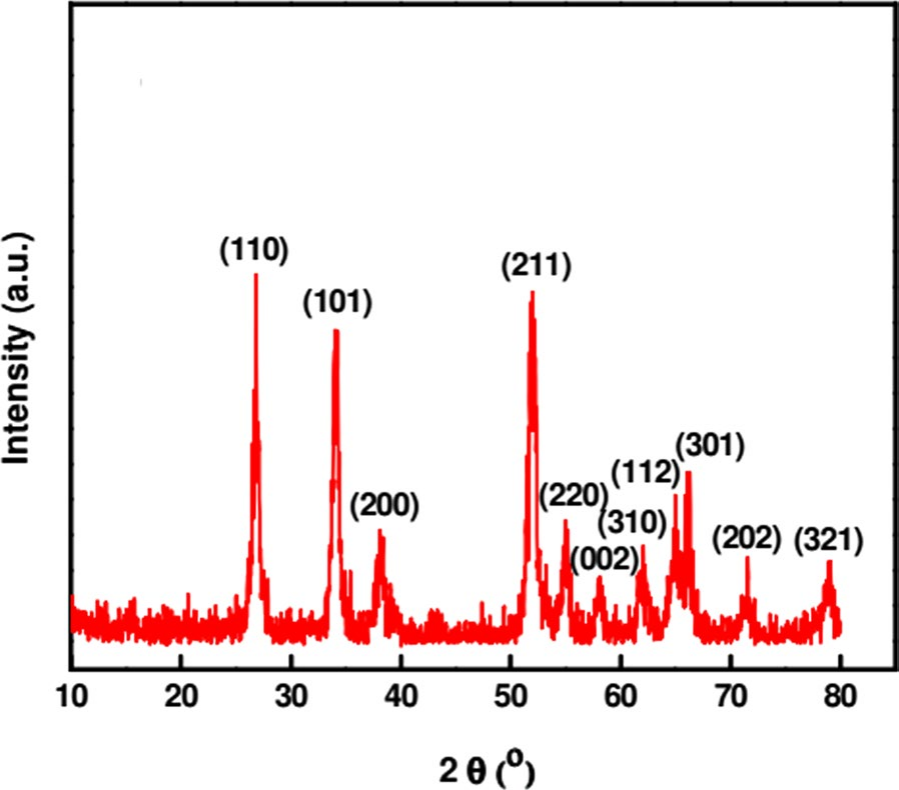
XRD patterns of SnO_2_ NPs synthesized using arginine under microwave irradiation [81]

Characterization of NPs is very crucial to know and control the synthesis procedure and application. The surface morphology and conformational details about size, shape, crystallinity, and surface area of the synthesized NPs are studied by utilizing various techniques. Some of the techniques used for characterizing green synthesized SnO_2_ NPs are UV–Vis, FTIR, XRD, EDS, SEM, and TEM. UV–Vis spectroscopy is used to monitor nanoparticle formation by studying their optical properties. UV–Vis spectra of the SnO_2_ NPs synthesized using *Catunaregam spinosa* extract exhibited the highest absorption peak of 223 nm due to its surface Plasmon resonance [57]. XRD is a powerful technique used for studying the crystal structure of materials. Bhattacharjee et al. [81] characterized SnO_2_ NPs with XRD peaks at diffraction angles (2*θ*) of 26.7, 34.2, 38.07, 51.9, 54.9, 58.1, 62.1, 64.9, 66.08, 71.6, and 79.07 corresponding to (110), (101), (200), (211), (220), (002), (310), (112), (301), (202), and (321) planes, respectively (Fig. 3). The standard diffraction peaks show the tetragonal rutile structure of SnO_2_ NPs with an average crystallite size 4.6 nm calculated using the Debye–Scherer equation [81].

**Fig. 4:**
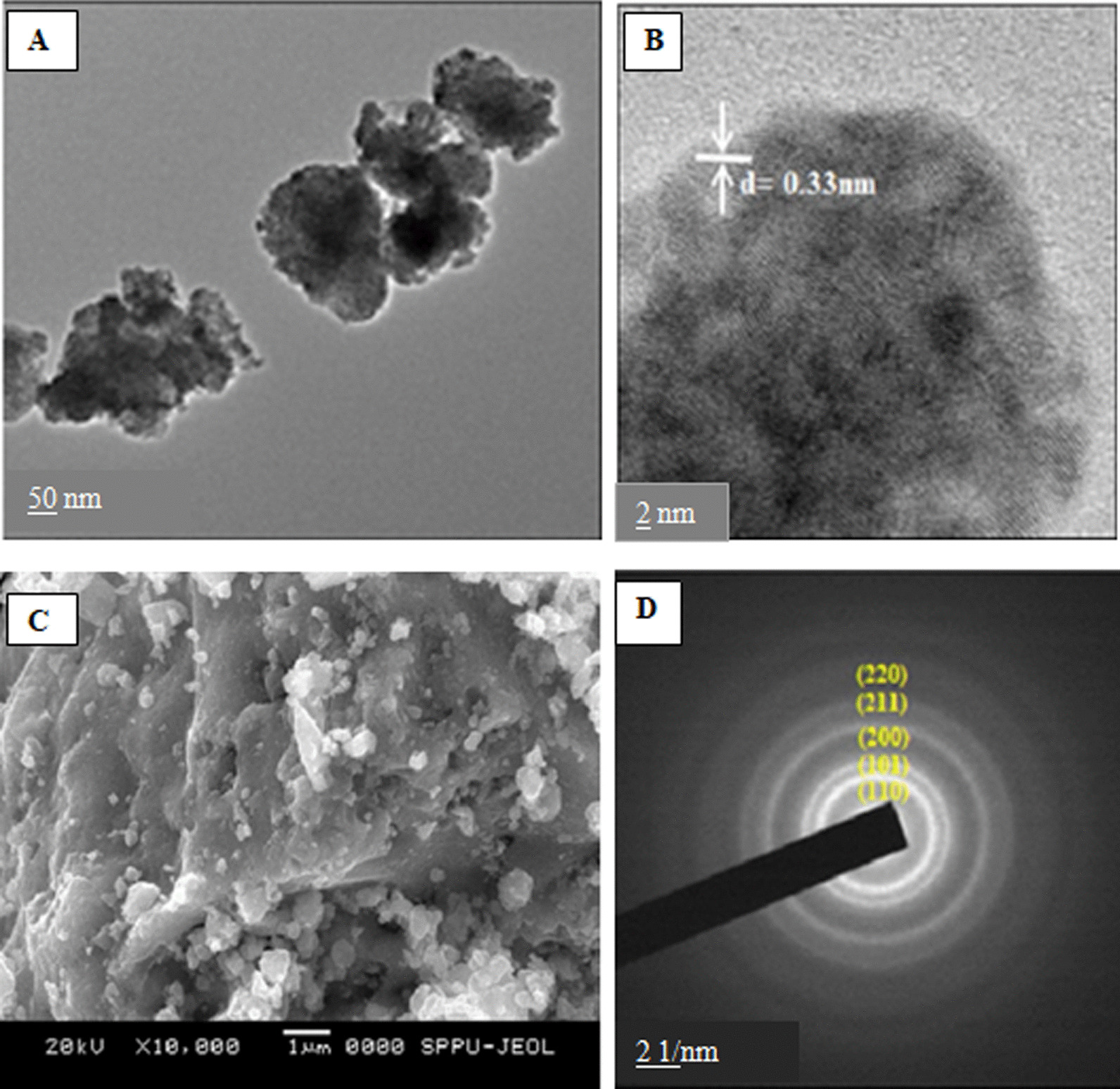
**a** TEM images of biosynthesized SnO_2_ NPs; **b** HR-TEM images of SnO_2_ NPs; **c** FE-SEM images of SnO_2_ NPs and **d** SAED patterns of SnO_2_ NPs

FTIR spectroscopy is used to investigate the surface chemistry and identify the functional groups present on the surface of the NPs, which might be responsible for reduction, capping, and stabilization of NPs [88]. FTIR analysis of SnO_2_ NPs synthesized using *Pruni flos* extract showed major absorption bands at 3308, 2153, 1634, 423, 403, and 383 cm^−1^. The strong bands observed at 3308, 2153, and 1634 cm^−1^ have been assigned to stretching vibrations of Sn–OH groups due to water molecules adsorbed on the SnO_2_ surface, C–H stretching of alkynes, and C=O vibrations of flavonoids, respectively. The bands between 423 and 383 cm^−1^ are attributed to anti-symmetric Sn–O–Sn stretching [70]. Microscopy-based techniques such as SEM and TEM have been widely employed to determine the morphological properties of the nanoparticles. However, TEM gives better resolution and information on the internal structure, such as crystal structure and morphology, as compared to SEM. The more accurate results of the surface properties can be acquired by using the FE-SEM. These techniques are also useful in estimating the average size of the synthesized nanoparticles [89]. FE-SEM and TEM analysis revealed the formation of slightly agglomerated spherical-shaped SnO_2_ NPs with an average size of 30–40 nm (Fig. 4) [62].

Furthermore, selective area electron diffraction (SAED) analysis showed the particles are nanocrystalline in nature (Fig. 4) [62]. The formation of spherical shape SnO_2_ NPs synthesized using *Piper betle* aqueous extract was ascertained by FE-SEM [63]. From TEM and XRD analysis, the average size of the NPs was found to be 8.4 nm.

EDS is an analytical technique used to analyze the elemental composition of a sample. The EDS spectra of SnO_2_ NPs synthesized using *Psidium Guajava* leaf extract revealed the presence of Sn and O peaks, which confirms the formation of pure SnO_2_ NPs [61].

## Biological Application of Green Synthesized Tin Oxide Nanoparticles

Green synthesized SnO_2_ NPs showed enhanced photocatalytic, antimicrobial, antioxidant, and anticancer activity as compared to their bulk form. In this section, we have discussed the applications of green synthesized SnO_2_ NPs in various fields as guidance to new researchers for future prospects.

### Antimicrobial Activity

Many researchers have observed antimicrobial activity SnO_2_ NPs. For instance, the antibacterial activity of SnO_2_ NPs synthesized using *aloe vera* plant extract was studied using *E. coli* and *S. aureus*. It was suggested that the NPs were more active against *S. aureus* than *E. coli* [58]. This may be because the cell wall of *E. coli* is more complex than *S. aureus*. *S. aureus* has a membrane composed of thick peptidoglycan. However, the cell wall of *E. coli* has peptidoglycan layer plus an outer membrane composed of lipopolysaccharide. The outer membrane of *E. coli* acts as a barrier that lowers the penetration level of ROS into the cell [90–92]. Another reason could be the difference in the polarity of their cell membrane. The membrane of *S aureus* has a more positive charge than *E. coli*, which result in greater penetration level of negatively charged free radicals causing more cell damage and death in *S. aureus* than in *E. coli* [93, 94]*.* The antibacterial activity of SnO_2_ NPs synthesized using *Punica granatum* seed extract has been tested against *E. coli.* The bactericidal effect increased with increasing the concentration of the nanoparticles [95]. SnO_2_ NPs synthesized using *Trigonella foenum-graecum* seed extract also showed similar antibacterial activity against *E. coli* [72]. In a recent study, *Clerodendrum inerme* leave extract was used for the synthesis of un-doped and Co-doped SnO_2_ NPs [96]. The green synthesized un-doped and Co-doped SnO_2_ NPs were subjected toward antimicrobial activity against five disease-causing pathogens such as *E. coli*, *B. subtillis*, *A. niger*, *A. flavus*, and *C. Albicans,*, and their zone of inhibition diameters (ZOIs), minimum inhibitory concentration (MIC), and minimum bactericidal concentration (MBC) were calculated. The Co-doped SnO_2_ NPs showed substantial antibacterial activity against *E. coli* and *B. subtillis* in a concentration-dependent manner compared to the un-doped SnO_2_ NPs, plant extract, and standard drugs in terms of their MIC (22 ± 0.7, 18 ± 0.8 mg/mL) and MBC (31 ± 0.9, 21 ± 0.6 mg/mL) as well as ZOIs (30 ± 0.08, 26 ± 0.06 nm), respectively. The authors suggested that the broad-spectrum antibacterial activity of Co-doped SnO_2_ NPs is due to cobalt doping, which leads to increased grain size and surface area of the NPs as compared to un-doped SnO_2_ NPs. As more is the surface area with smaller particle sizes, the greater will be the antimicrobial activity. The associations of biomolecules like flavonoids and phenolic compounds with Co-doped SnO_2_ NPs are also responsible for the enhancement of their antimicrobial activity. Furthermore, the green synthesized Co-doped SnO_2_ NPs showed extraordinary antifungal activity with maximum ZOIs of 17 ± 0.04, 23 ± 0.08, and 26 ± 0.06 nm against *A. niger*, *A. flavus,* and *C. Albicans,* respectively, in comparison with plant extract, un-doped SnO_2_ NPs, and standard drugs [96].

The actual mechanism of action of SnO_2_ NPs against microbial strains is still unknown. However, several mechanisms of action against bacteria have been suggested for metal oxide nanoparticles, such as the decomposition of nanoparticles, electrostatic interaction of nanoparticles with the cell wall of microorganisms, and formation of reactive oxygen species (ROS) by the effect of light radiation [97–99]. One possible cause for the antibacterial effect of SnO_2_ NPs may be the accumulation of the NPs on the surface of the bacterial cell membrane. The ROS generated due to the presence of SnO_2_ NPs interacts with the cell membrane and disturbs the membrane permeability and respiration system of the bacteria, which leads to cell death [72, 95, 100]. For instance, Khan et al. suggested that the release of Sn^4+^ and Co^2+^ is responsible for the damage of bacterial DNA and mitochondria, which inactivates the bacterial enzyme and finally leads to cell death [96].

### Antioxidant Activity

Many researchers examined the antioxidant activity of NPs by monitoring the ability in quenching of stable DPPH (2,2-diphenyl-1-picrylhydrazyl) radical into non-radical form (DPPH-H). Kamaraj et al. [101] reported the antioxidant activity of SnO_2_ NPs biosynthesized using *Cleistanthus Collinus* leaves extract. The antioxidant activity of SnO_2_ NPs increased with increasing concentration of SnO_2_ NPs and reaction time. In another study [95], the free radical scavenging activity of green synthesized SnO_2_ NPs increased in a dose-dependent manner. However, the annealed sample exhibited a lower scavenging activity as compared to the as-prepared sample. The decrease in scavenging activity with increasing annealing temperature may be due to a decrease in surface area-to-volume ratio of the NPs. Moreover, the antioxidant efficacy of SnO_2_ NPs against DPPH is due to the transfer of electron density between the NPs and the free radical located at nitrogen in DPPH. A similar result was found for SnO_2_ NPs synthesized using *Trigonelle foenum-graecum* aqueous extract [72]. Hong et al. [67] reported significant antioxidant properties of biosynthesized SnO_2_ NPs. The antioxidant activity of the biosynthesized SnO_2_ increased with increasing concentration of the NPs, with IC_50_ (half-maximal inhibitory concentration) value of 2257.4 µg/ml. Recently, Khan et al*.* reported significant antioxidant activity of Co-doped SnO_2_ NPs, as compared to un-doped SnO_2_ NPs and plant extract [96].

### Cytotoxic Activity

**Fig. 5:**
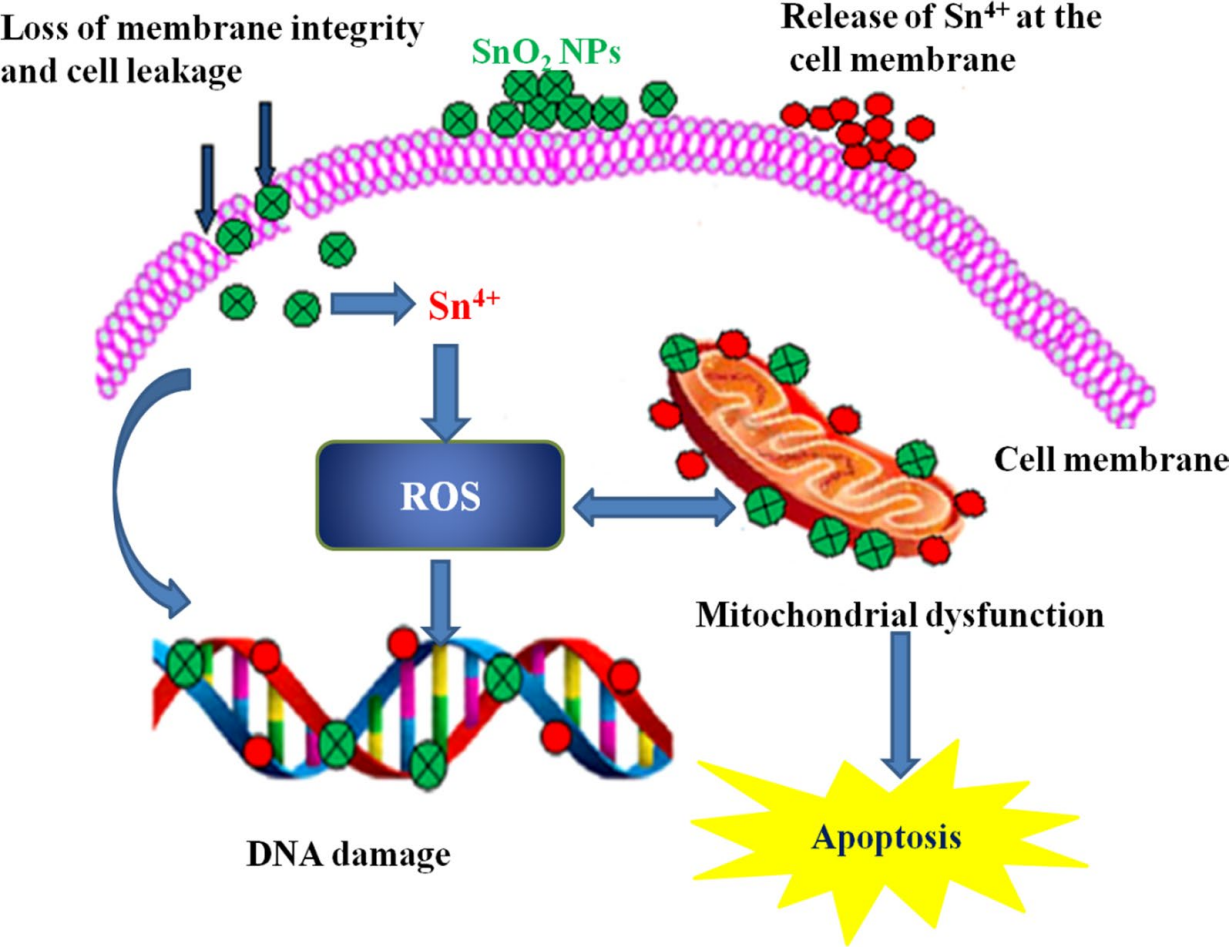
Mechanism during cytotoxicity of green synthesized SnO_2_ NPs

SnO_2_ NPs synthesized using an aqueous extract of agricultural waste of dried peel of *Annona squamosa* were evaluated for cytotoxicity test against the hepatocellular carcinoma cell line (HepG2) [64]. TEM results revealed the loss of cell volume, considerable swelling of the cells, and nuclear condensation. The nuclear condensation seen in SnO_2_ NP-treated HepG2 cells might be due to the breakdown of chromatin in the nucleus. SnO_2_ NPs inhibited cell proliferation in a dose- and time-dependent manner with an IC_50_ value of 148 µg/mL. SnO_2_ NPs synthesized using *piper nigrum* seed extract exhibited higher cytotoxic activity against colorectal (HCT116) and lung (A549) cancer cell lines [69]. The proliferation of both cancer cell lines increased with increasing NPs size. Besides, a gradual decline in cell viability was observed with increasing dosage of SnO_2_ NPs. The authors concluded that the cytotoxic effect was associated with the generation of oxidative stress from reactive oxygen species (ROS). SnO_2_ NPs fabricated by using *Pruni spinosae flos* aqueous extract revealed an excellent cytotoxic effect against non-small cell lung cancer cell A549 and lung fibroblast CCD-39Lu cells, in concentration- and time-dependent manner [70]. Recently, Khan et al. [96] investigated the in vitro cytotoxic effect of green synthesized un-doped SnO_2_ NPs and Co-doped SnO_2_ NPs against mammary gland breast cancer (MCF-7), human amnion (WISH), and human lung fibroblast (WI38) cell lines by a colourimetric technique 3-(4,5-dimethylthiazol-2yl)-2,5-diphenyl tetrazdium bromide (MTT) assay. The green synthesized Co-doped SnO_2_ NPs showed significant and substantial mortality rate compared to un-doped SnO_2_ NPs, plant extract, and standard drug 5-FU (5-5-5-fluorouracil), while un-doped SnO_2_ NPs exhibited a similar mortality rate to that of the standard drug, but the lowest cytotoxicity against breast cancer cell line was observed with the plant extract. The cytotoxic effect of the plant extract, green synthesized un-doped SnO_2_, and Co-doped SnO_2_ NPs was performed with IC_50_ values of 31.56 ± 1.4, 26.99 ± 1.9, and 18.15 ± 1.0 µg/mL for MCF-7, and 40.69 ± 0.9, 38.97 ± 0.8, and 36.80 ± 0.6 µg/mL for WI38, and 38.56 ± 0.8, 35.56 ± 0.9, and 31.10 ± 0.7 µg/mL, respectively, and depicted that the cytotoxicity of Co-doped SnO_2_ NPs was more proficient as compared to plant extract and un-doped SnO_2_ NPs alone. Additionally, the authors reported excellent observational results showing greater in vitro inhibition of breast cancer MCF-7 cell lines in a concentration- and dose-dependent manner. The green synthesized NPs also showed robust cytotoxicity against MCF-7 as compared to WI38 and WISH normal cell lines with Co-doped SnO_2_ NPs unveiling higher ROS generation than the un-doped SnO_2_ NPs. Figure 5 shows the probable cytotoxicity mechanism of green synthesized SnO_2_ NPs.

### Photocatalytic Activity

**Fig. 6:**
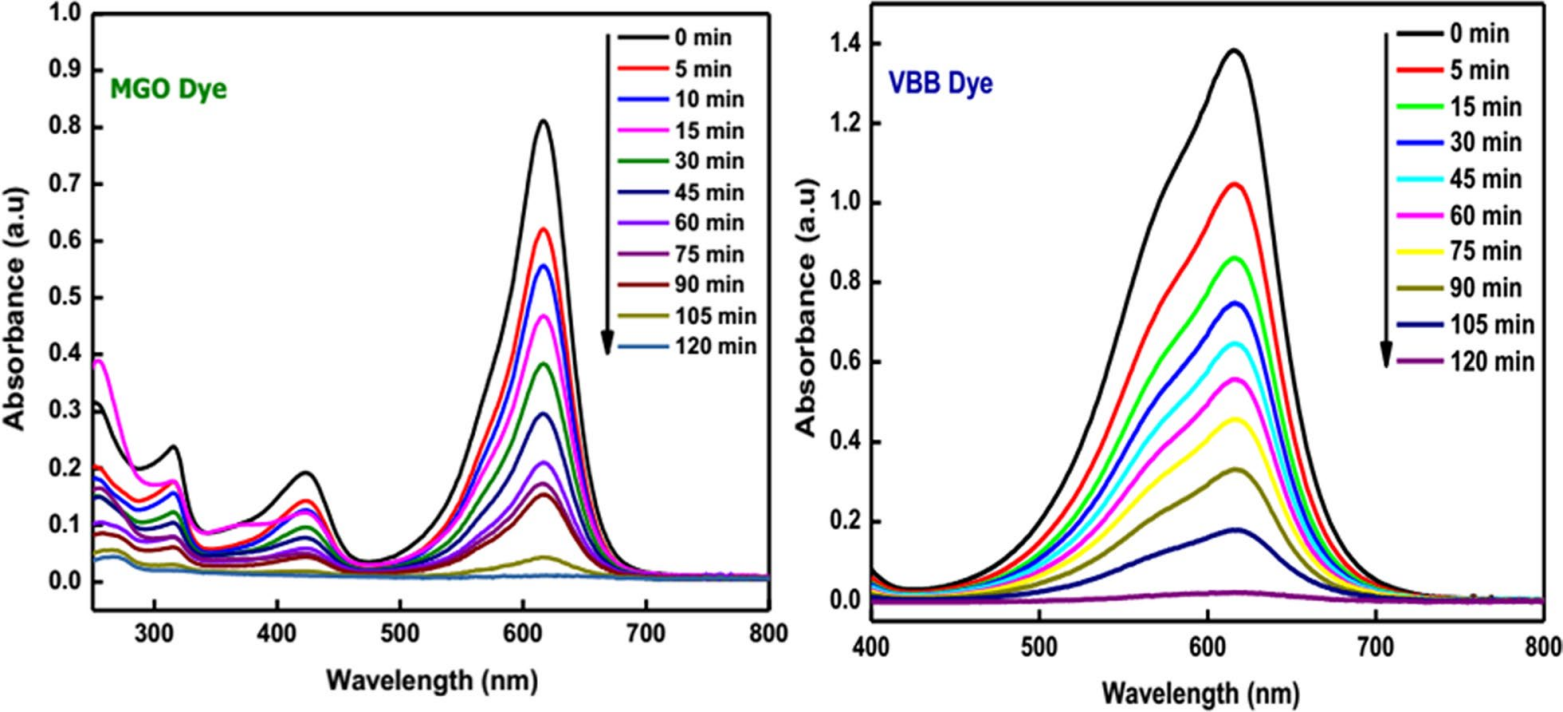
Photocatalytic degradation of malachite green oxalate dye under solar irradiation using SnO_2_ NPs

The uncontrolled release of toxic chemicals, hazardous textile dyes, and pesticides from various industries into the running water has led to severe environmental problems. These superfluous water contaminants cause long-term adverse effects and pose a real threat to aquatic and human life. Besides, some organic dyes are carcinogenic and toxic. Hence, treating water that contains poisonous chemicals before disposal to the environment is very crucial to reduce environmental pollution. Recent studies have shown that nanostructured semiconductor metal oxides act as an excellent photocatalyst for the removal of various water pollutants [102–105]. Among the semiconductor metal oxides, SnO_2_ is extensively used in the removal of common textile dyes and organic compounds owing to its beneficial characteristics, which include physical and chemical stability, high surface reactivity, high photocatalytic efficiency, low cost, and low toxicity [68, 106]. Manjula et al. [107] synthesized SnO_2_ nanoparticles using glucose. The NPs were effectively used as a catalyst in degrading methyl orange (MO) dye. The effect of calcination temperature (150–500 °C) on the photocatalytic activity of the NPs was investigated, and the results revealed that the as-synthesized SnO_2_ NPs calcinated at 150 °C is the best photocatalyst for the reaction under study among the studied materials. Moreover, the as-prepared SnO_2_ nanoparticles degraded methyl orange completely in 30 min, and also, the nanomaterials may be recycled with enhanced efficiency a minimum of five times. In another study [108], the photocatalytic activity of the SnO_2_ QDs (quantum dots) synthesized by using serine was evaluated by monitoring the optical absorption spectra of eosin Y solution under direct sunlight. It was observed that the rate of degradation of eosin Y using SnO_2_ QDs (98%) is higher than that using commercial SnO_2_ (96%) and P25 (88%). Begum et al. [84] reported the synthesis of SnO_2_ NPs using an amino acid, L-lysine monohydrate. The synthesized nanoparticles were evaluated for their photocatalytic behavior toward toxic organic dyes, namely malachite green oxalate (MGO) and Victoria blue B (VBB) under direct sunlight. The absorption peak of these dyes has begun to reduce, which shows that the chromophore structure has been demolished (Fig. 6). Furthermore, the as-prepared SnO_2_ NPs exhibited an outstanding photocatalytic degradation of MGO (97.3%) and VBB (98%) dye within 120 min. Literature reports on the photocatalytic activity of green synthesized SnO_2_ NPs are summarized in Table 3.

**Table 3:** Photocatalytic activity of SnO_2_ NPs synthesized using various biological entities

Source used for SnO_2_ NPs synthesis	Dyes used for analysis	Size of NPs (nm)	Time (min)	Degradation (%)	Ref
*Brassica oleracea L. var. botrytrs*	Methylene blue	3.6–6.3	180	92	[52]
*Calotropis gigantea*	Methyl orange	35	180	80	[62]
*Catunaregam spinosa*	Congo red	47	20	92	[57]
*Persia Americana*	Phenolsulfonphthalein	4	120	100	[68]
*E. herbicola*	Methylene blue	10–42	120	93.3	[54]
	Erichrome black T		120	97.8	[54]
	Methyl orange		120	94.0	[54]
Tyrosine	Violet 4BSN	15–20	30	100	[85]
L-lysine	Malachite green oxalate	4	120	97.5	[84]
	Victoria blue B		120	98	[84]
Arginine	Methylene blue	4–5	240	94	[81]
Glucose	Methyl orange	50	30	100	[107]

### Gas-Sensing Property

Many metal oxide-based gas sensors are widely used for gas-sensing applications. However, SnO_2_ NPs has gained tremendous attention in gas sensing under atmospheric conditions because of its beneficial properties, which include high sensitivity, high selectivity, easy reversibility, and cheap manufacturing costs. Green synthesized porous SnO_2_ nanospheres demonstrated excellent gas sensing capabilities [107]. It was observed that the assimilation of 0.5 wt% Pd into the SnO_2_ matrix improved the sensitivity and made it highly selective for low-temperature hydrogen detection. Moreover, the fabric was able to respond to even 50 ppm H_2_ in N_2_ at room temperature with an interval of 10 s. These sensing properties are due to the synergetic effect of both the porous structure of SnO_2_ nanospheres and also the catalytic property of Pd nanoparticles. Gattu et al*.* [77] reported the gas-sensing behavior of biosynthesized and chemically synthesized Ni-doped SnO_2_ NPs thin films. The biosynthesized Ni-doped SnO_2_ NPs thin film showed higher NO_2_ gas-sensing response as compared to the chemically synthesized ones. The sensor response was found to be increased with Ni doping for both biosynthesized and chemically synthesized Ni-doped SnO_2_ NPs. This may be due to the reduction of particle size with Ni-doping, which results in increased surface area for adsorption of NO_2_ gas. Furthermore, the Ni-doped SnO_2_ thin film exhibited excellent selectivity toward NO_2_ gas when put next to other gases like NH_3_, LPG and H_2_S. In another study [78], the gas-sensing properties of un-doped and Fe-doped SnO_2_ NPs synthesized using *Cicer arietnum* L. extract were reported. The gas response within the presence of 100 ppm NH_3_ gas at 200 °C operating temperature was found to be 28% for un-doped SnO_2_ and 46% for Fe-doped SnO_2_ thin films. Moreover, the Fe-doped SnO_2_-based sensor was found to be more selective for NH_3_ gas as compared to the un-doped SnO_2_ sensor. The biosynthesized Au-doped SnO_2_ NPs were found to be highly sensitive to NO_2_ gas at 200 °C operating temperature [79]. Gas sensor supported Au-doped SnO_2_ NPs showed the gas response of ~ 30% for 100 ppm of NO_2_ gas. Additionally, the gas sensor of Au-doped SnO_2_ NPs showed excellent selectivity toward NO_2_ gas when put next to other gases like H_2_S, LPG, and NH_3_. The improved gas response and selectivity toward NO_2_ gas are because of the lattice distortion induced by Au-doping and also the oxygen vacancies generation within the SnO_2_ lattice.

## Conclusions

The use of green methods for the production of NPs has been the area of focused research because it is an eco-friendly, inexpensive, nontoxic, and sustainable method. Numerous studies report the possibility of producing SnO_2_ NPs via a green protocol using a range of plant materials, bacteria, and natural biomolecules. The literature survey shows that the green substrates act as reducing and stabilizing agents or capping agents regardless of their source. Among the various green methods of SnO_2_ synthesis, plant-mediated synthesis is cost-effective, easy to process, and less hazardous than microorganisms. However, the plant extracts consist of a large number of active compounds in a different composition, which makes it difficult to know the exact amount of the molecules responsible for the reduction of metal ions. Due to this complexity, it is difficult to evaluate the synthesis of nanoparticles. Therefore, further study on the mechanism of formation of SnO_2_ NPs is required to understand the chemical reactions that occur during the synthesis. With the knowledge of the actual reaction mechanism, it will be possible to monitor and optimize the biosynthesis process, which is essential for the large-scale production of SnO_2_ NPs. Hence, understanding of the rapidly growing method of synthesis discussed herein will help facilitate future research progress on SnO_2_ NPs and their enormous potential for industrial-scale production in the near future.

## Data Availability

Not available.

## Abbreviations

NPs: Nanoparticles
SnO_2_ NPs: Tin oxide nanoparticles
UV-Vis: Ultraviolet-visible spectroscopy
SEM: Scanning electron microscopy
FE-SEM: Field emission scanning electron microscopy
TEM: Transmission electron microscopy
HR-TEM: High-resolution transmission electron microscopy
FTIR: Fourier transform infrared spectroscopy
EDS: Energy dispersive X-ray analysis
PSA: Particle size analyzer
XPS: X-ray photoemission spectroscopy
SAED: Selective area electron diffraction
